# Cytokine signature in convalescent SARS-CoV-2 patients with inflammatory bowel disease receiving vedolizumab

**DOI:** 10.1038/s41598-023-50035-1

**Published:** 2024-01-02

**Authors:** Simone Dallari, Vicky Martinez Pazos, Juan Munoz Eusse, Judith Wellens, Craig Thompson, Jean-Frederic Colombel, Jack Satsangi, Ken Cadwell, Serre-Yu Wong, ICARUS-IBD Working Group, Jessica Anne Neil, Stela Sota, Kyung Ku Jang, Krystal Ching, Mericien Venzon, Xiaomin Yao, Lucie Bernard, Xin Chen, Michael Tankelevich, Reema Navalurkar, Rebekah Dixon, Drew S. Helmus, Marcia Mukanga Lange, Emily Spiera, Lodoe Sangmo

**Affiliations:** 1 Department of Microbiology, New York University Grossman School of Medicine, New York, USA; 2 The Henry D. Janowitz Division of Gastroenterology, Icahn School of Medicine at Mount Sinai, One Gustave L. Levy Place, Box 1069, New York, NY 10029 USA; 3 Icahn School of Medicine at Mount Sinai, New York, USA; 4 Department of Gastroenterology and Hepatology, Leuven University Hospitals, Leuven, Belgium; 5 Division of Biomedical Sciences, Warwick Medical School, University of Warwick, Warwick, UK; 6 Nuffield Department of Medicine, Translational Gastroenterology Unit, University of Oxford, Oxford, UK; 7 Division of Gastroenterology and Hepatology, Department of Medicine, Department of Systems Pharmacology and Translational Therapeutics, Department of Pathology and Laboratory Medicine, University of Pennsylvania Perelman School of Medicine, Philadelphia, PA USA; 8 University of Washington, Washington, USA

**Keywords:** Inflammatory bowel disease, Viral infection

## Abstract

While differential antibody responses SARS-CoV-2 in patients with inflammatory bowel disease (IBD) receiving infliximab and vedolizumab are well-characterized, the immune pathways underlying these differences remain unknown. Prior to COVID-19 vaccine development, we screened 235 patients with IBD receiving biological therapy for antibodies to SARS-CoV-2 and measured serum cytokines. In seropositive patients, we prospectively collected clinical data. We found a cytokine signature in patients receiving vedolizumab who are seropositive compared with seronegative for SARS-CoV-2 antibodies that may be linked to repeated SARS-CoV-2 infections. However, there were no differences between seropositive and seronegative patients receiving infliximab. In this single-center cohort of patients with IBD with anti-SARS-CoV-2 antibodies at the onset of the COVID-19 pandemic, and therefore without influence of vaccination, there is a cytokine signature in patients receiving vedolizumab but not infliximab. These findings lay the groundwork for further studies on immune consequences of viral infection in patients with IBD, which is postulated to evolve from aberrant host-microbe responses.

## Introduction

Differential effects of biological therapies on immune response to SARS-CoV-2 are of significant interest to patients with IBD and their providers. The CLARITY-IBD study reported that IBD patients receiving vedolizumab mount higher antibody responses to SARS-CoV-2 infection and vaccination compared with those receiving infliximab^1–3^. Subsequent studies have described functional antibody responses and cellular responses to SARS-CoV-2 in IBD patients receiving biological therapies^4^. However, the immune pathways underlying these biological treatment effects on antibody responses to SARS-CoV-2 infection remain undefined.

## Results

We asked what circulating immune and inflammatory mediators are associated with antibody responses to SARS-CoV-2 infection in patients with IBD receiving biological therapies. For the New York City cohort of ICARUS-IBD, a multinational study of longitudinal serological responses to SARS-CoV-2, we evaluated the presence of antibodies to SARS-CoV-2 and levels of circulating cytokines^5,6^. To ensure that serological measurements reflect exposure to SARS-CoV-2 virus within four months and not COVID-19 vaccination, we utilized data and samples collected from visits occurring between 26 May and 15 July 2020 (n = 235) (Table 1). Most cases in this study were not confirmed due to lack of available testing for patients who did not require urgent care visits or hospitalization during that time period. The first documented case of COVID-19 in New York City was 1 March 2020 with lockdown beginning 16 March 2020. Seropositive patients in this study reported symptoms from late February through April 2020. Given this, the estimated time period between infection and sampling was between 1 and 4 months. Patients who were seropositive for anti-SARS-CoV-2 Spike (S) antibodies were followed through chart review and phone calls to patients in September 2022, of whom 11 of 21 patients responded.

**Table 1:** Patient characteristics of SARS-CoV-2 seropositive patients with IBD in the study.

Age	Sex	IBD type	Medication	Comorbid conditions	Episodes of COVID-19	COVID severity 1	COVID severity 2	COVID severity 3	Vaccine type	Long COVID symptoms	Cytokine cluster
20	F	CD	Infliximab	None	2	Asymptomatic	Mild	n/a	Pfizer (3 doses)	Unknown	2
19	M	CD	Infliximab	Growth failure	1	Asymptomatic	n/a	n/a	Unknown	Unknown	1
20	M	CD	Infliximab	Obesity	1	Asymptomatic	n/a	n/a	Pfizer (2 doses)	Unknown	0
27	M	CD	Infliximab	None	1	Mild symptoms in March 2020	n/a	n/a	J&J (1 dose)	Unknown	1
40	M	CD	Infliximab	Hypertension	1	Asymptomatic	n/a	n/a	None	None	–
44	M	CD	Infliximab	Obesity, Asthma, Hypertension	3	Mild symptoms in March/April 2020	Mild	Mild	Pfizer (3 doses)	Unknown	0
65	M	CD	Infliximab	None	1	Unknown	n/a	n/a	Unknown	Anosmia	0
27	M	UC	Infliximab	Obesity	1	Mild symptoms March 2020	Asymptomatic	n/a	J&J	None	–
57	M	UC	Infliximab + 6-mercaptopurine	Hypertension, DM, HCV	1	Mild symptoms in March/April 2020	n/a	n/a	Pfizer	Tinnitus	1
75	F	UC	Infliximab	None	1	Mild disease March 2020	n/a	n/a	Moderna (3 doses)	None	–
29	M	IBD-U	Infliximab	None	1	Asymptomatic	n/a	n/a	Pfizer (3 doses)	Unknown	2
62	F	CD	Ustekinumab	Lupus	1	Mild disease April 2020	n/a	n/a	J&J	None	–
26	M	CD	Ustekinumab	None	1	Mild symptoms February 2020	n/a	n/a	Pfizer (2 doses)	Unknown	2
39	M	CD	Vedolizumab	None	1	Mild disease March 2020	n/a	n/a	Unknown	None	–
70	M	CD	Vedolizumab	HIV	2	Mild disease March 2020	Mild	n/a	Moderna (3 doses)	Upper extremity neuropathy	0
85	M	CD	Vedolizumab	Hypertension	3	Asymptomatic	Moderate	Mild	Moderna (3 doses)	Unknown	0
27	M	UC	Vedolizumab + methotrexate (discontinued mid-way through study)	None	2	Asymptomatic	Mild	n/a	Moderna (4 doses)	None	0
34	M	UC	Vedolizumab	Obesity	3	Mild symptoms April 2020	Mild	Mild	Pfizer (3 doses)	Throat discomfort, brain fog, anxiety	0
46	M	UC	Vedolizumab	HIV	2	Hospitalized with severe disease April 2020	Moderate	n/a	Pfizer (3 doses)	Lower extremity weakness	0
72	M	UC	Vedolizumab	Lipid disorder	1	Hospitalized with severe disease March 2020	n/a	n/a	Pfizer (3 doses), Moderna (1 dose)	Unknown	–
77	M	UC	Vedolizumab	None	1	Unknown	n/a	n/a	Unknown	Unknown	0

**Figure 1:**
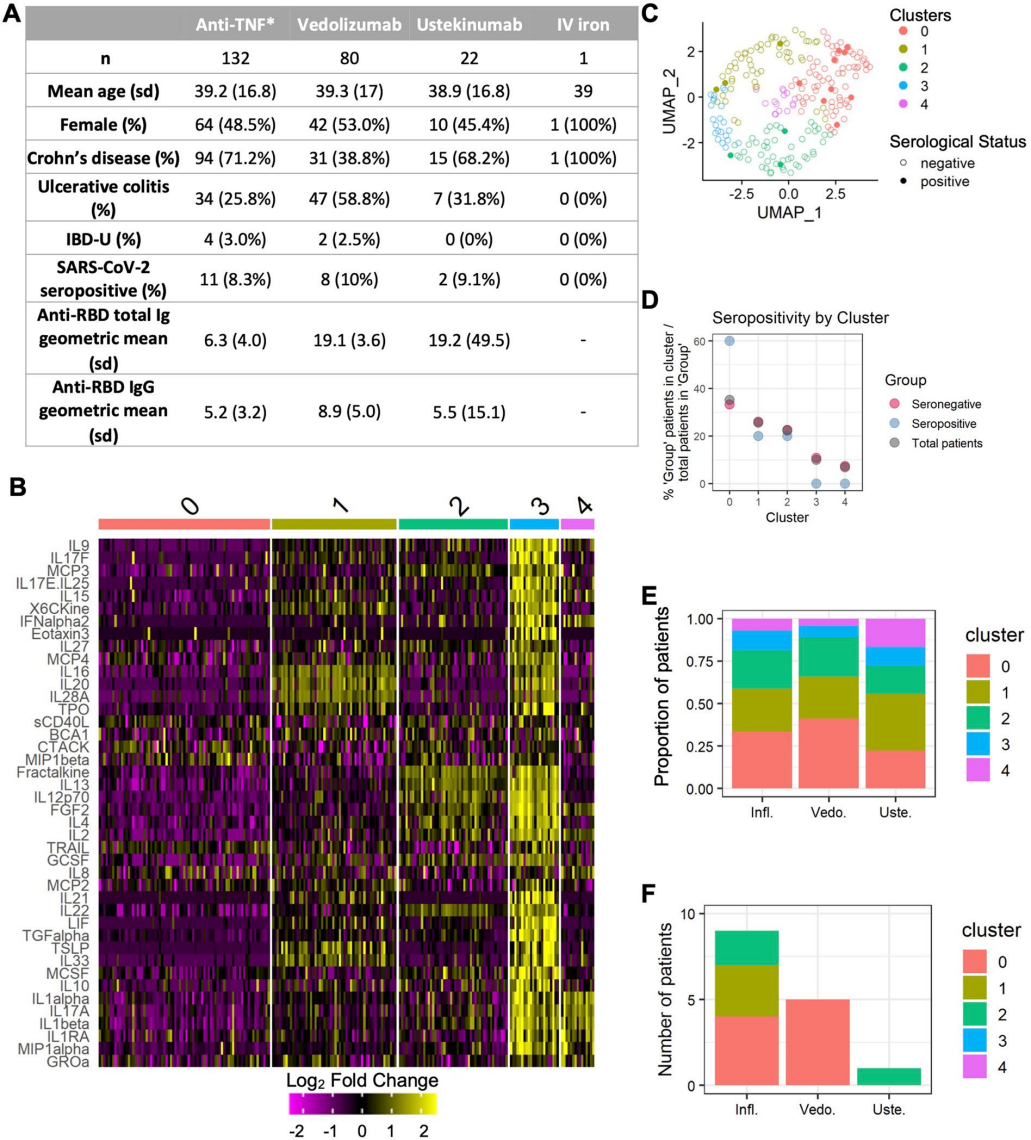
Cytokine array reveals unique clustering of IBD patients by presence or absence of antibodies to SARS-CoV-2. (**A**) Patient characteristics and antibody results of 235 IBD patients screened for antibodies to SARS-CoV-2. *One patient received certolizumab pegol. All other patients received infliximab. (**B**) Heatmap of cytokine array results showing the top ten cytokines associated with each cluster. (**C**) UMAP plot of patients by cluster and SARS-CoV-2 sero-status. (**D**) Graph showing distribution of total and SARS-CoV-2 seronegative and seropositive patients in clusters 0–5 by percentage of patients within each group, respectively. (**E**) Cluster distribution of all patients (**E**) and seropositive patients alone (**F**) by medication.

Of 21 patients (8.9%) who tested positive for anti-SARS-CoV-2 antibodies, the anti-S levels in seropositive patients receiving infliximab were lower than in those receiving vedolizumb, consistent with previous studies (p < 0.0001) (Fig. 1A). Of available clinical data, most patients had asymptomatic or mild infections (infliximab n = 10/10, ustekinumab 2/2, vedolizumab n = 5/7). The two patients hospitalized with severe COVID were both receiving vedolizumab (*n* = 2/21) (Table 1). Of infliximab patients, 29%% (2/11) had greater than 1 infection compared with 63% (5/9) patients receiving vedolizumab, though this was not statistically significant (p = 0.07). Long COVID symptoms, as defined by the Centers for Diseases and Prevention website, were reported by 40% (2/5) of infliximab, 0% (0/1) of ustekinumab, and 60% of vedolizumab (3/5) patients (p > 0.05).

Levels of cytokines and chemokines from all patients were measured by the Eve Technologies HD71 assay. After data normalization and exclusion of proteins with undetectable levels across samples, we performed unbiased clustering analysis on levels of all cytokines (Fig. 1B–E). Antibody formation to SARS-CoV-2 accounted for less than 1% of the variance. However, seropositive patients were highly represented in Cluster 0, which contains 60% of the SARS-CoV-2 seropositive patients whereas clusters 3 and 4 contain no seropositive patients (Fig. 1B–D). All seropositive vedolizumab patients were in Cluster 0, whereas seropositive infliximab patients were distributed amongst clusters 0, 1, and 2 (Fig. 1F). Notably, cluster 0 shows overall suppressed cytokine levels compared to other clusters.

**Figure 2:**
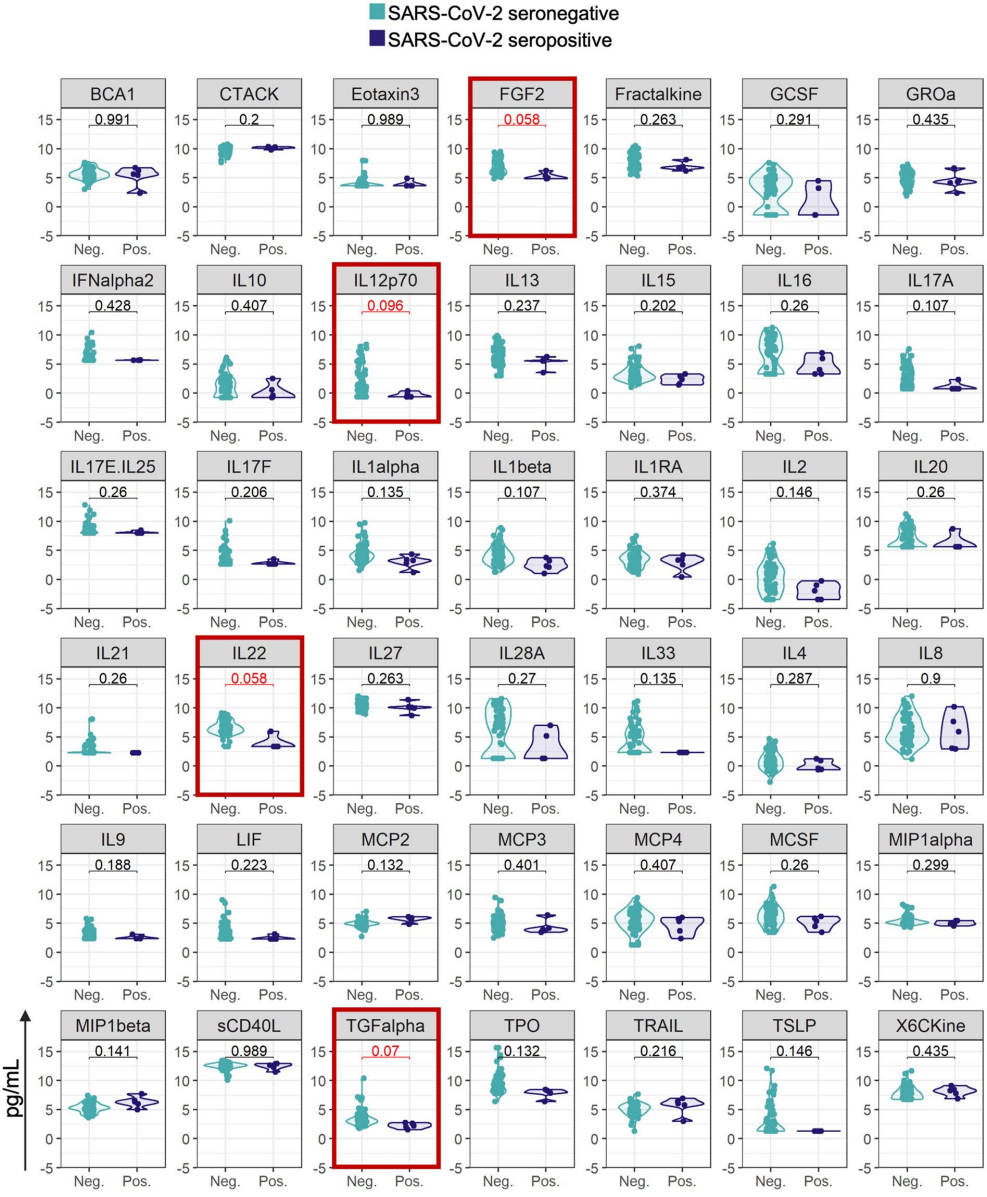
Violin plots comparing SARS-CoV-2 seropositive and seronegative patients receiving vedolizumab. Y-axis are log-transformed values of cytokine levels. Serum levels of the top 10 cytokines defining clusters 0 through 4 are plotted. FDR values are indicated on each graph. Red-outlined plots represent cytokines with an FDR < 0.1 between seronegative and seropositive patients within each medication group.

When we evaluated serum levels of the top ten cytokines defining each cluster in seronegative versus seropositive patients within each medication group, we found a signature in vedolizumab patients associated with seropositivity, including lower levels of FGF2 (fibroblast growth factor 2), interleukin (IL)-12p70 (a target of ustekinumab), IL22, and transforming growth factor (TGF)-α (Fig. 2). In contrast, in patients treated with the anti-tumor necrosis factor (TNF) infliximab there were no significant differences between cytokine profiles in seropositive versus seronegative patients (Supplemental Fig. 1).

Of longitudinal outcomes measured, there were seven patients who developed breakthrough infections, of whom six patients were in cluster 0 and one patient was in clusters 1 and 2 combined (p = 0.12) (Table 1). Of eleven patients who responded, five patients reported long COVID symptoms, of whom four were in cluster 0 and one was in clusters 1 and 2 combined (p = 0.08).

## Discussion

In summary, we demonstrate that, in IBD patients treated with vedolizumab who have convalesced from COVID-19, SARS-CoV-2 antibody responses are associated with a circulating cytokine signature distinct from seronegative patients and may be associated with long-term outcomes such as risk of recurrent SARS-CoV-2 infections. Cytokines that define this signature have been associated with COVID-19 responses in studies of non-IBD patients. IL12p70, a target of ustekinumab, and IL22, which is important for maintenance of intestinal homeostasis, were found in signatures associated with worsening COVID-19 severity^7,12^. FGF2 has previously been reported to be elevated in severe COVID-19^13^. TGFalpha has not been directly implicated in COVID-19, but blockade of signaling through its receptor, epidermal growth factor receptor, is linked to inhibition of SARS-CoV-2 replication^14^.

On the other hand, in IBD patients receiving anti-TNFs who have convalesced from SARS-CoV-2 infection, we found that the systemic cytokine milieu appears to remain stable and/or achieve homeostasis similar to that of SARS-CoV-2 seronegative patients. Moreover, while not statistically significant, fewer patients receiving anti-TNF reported long COVID symptoms than those receiving vedolizumab. In addition, pooled analysis from three major registries showed that treatment with anti-TNFs was inversely correlated with severe COVID-19 and death from COVID-19^15^. Based upon these observations, we postulate that anti-TNFs may help to limit perturbation to systemic immune homeostasis caused by SARS-CoV-2 infection, at least in comparison with gut-specific anti-integrin therapy (vedolizumab), thereby reducing severity of acute COVID-19 as well as long COVID. There may also be clinical significance to cluster 0, where seropositive patients in this cluster experienced more recurrent SARS-CoV-2 infections than those in other clusters independent of medication.

Limitations of this work are that it involves a small sample size, lacks controlled timing of samples from SARS-CoV-2 onset, and does not provide mechanistic data. Unfortunately, we did not have access to samples from non-IBD patients at our center, and significant batch effects precluded comparison with data from a study of non-IBD patients at a nearby center (Yale New Haven Hospital)^7^.

Nevertheless, we were able to capture de novo serum cytokine signatures of patients with IBD exposed to SARS-CoV-2 without the influence of vaccination. While studies in non-IBD patients have implicated T cell responses and cytokines such as IL-33 to be associated with asymptomatic infection and the convalescent phase of SARS-CoV-2 infection, to our knowledge this is the first study to similarly interrogate cytokine expression in patients with IBD^8–10^. Our data suggest that anti-integrin therapy differentially modulates the cytokine milieu and humoral response to SARS-CoV-2 infection compared with anti-TNFs. Vedolizumab may affect systemic immunity through altering the pool of circulating lymphocytes, such as through inhibiting their intestinal retention or their reactions against SARS-CoV-2 viral RNA that persist in the gut^11^.

While the effects of biological therapies on SARS-CoV-2 pathogenesis and the convalescent phase remain to be fully explored, our identification of cytokines expressed differentially according to infectious status in these patients lay the groundwork for future investigation of the immune effects of SARS-CoV-2 in patients with IBD.

## Methods

### Cohort

This was an observational cohort study conducted at the Mount Sinai Therapeutic Infusion Center in New York City from May 26 to July 15, 2020. The study protocol was approved by the Icahn School of Medicine at Mount Sinai Institutional Review Board and COVID-19 Research Committee (STUDY number 20-00527), and all research was performed in accordance with the Declaration of Helsinki and HIPAA regulations. Adults 18 years and older with scheduled appointments at the infusion center were eligible for recruitment. Written informed consent was obtained from eligible participants. Patient data including sex, infusion diagnoses, and IBD-related medications were obtained from medical records. An online questionnaire was administered to patients to self-report demographics, COVID-19 symptom history by month dating back to January 2020, and COVID-19 testing history. In September 2022, seropositive patients were contacted and administered a survey to report breakthrough infections, vaccinations, and long-COVID symptoms as per the Department of Health and Human Services and Centers for Disease and Prevention (https://www.covid.gov/longcovid/definitions and https://www.cdc.gov/coronavirus/2019-ncov/long-term-effects/index.html#:~:text=Long%20COVID%20is%20broadly%20defined,after%20acute%20COVID%2D19%20infection.). Regarding long COVID symptoms, patients were asked specifically if they had any of the following symptoms for at least 1 month: tiredness or fatigue that interferes with daily life, fever, difficulty breathing or shortness of breath, cough, chest pain, fast-beating or pounding heart (palpitations), neurological symptoms, difficulty thinking or concentrating, headache, sleep problems, dizziness upon standing (lightheadedness), pins-and-needles feelings, change in smell or taste, depression or anxiety, digestive symptoms, diarrhea, stomach pain, other symptoms, joint or muscle pain, rash, changes in menstrual cycles (if applicable), or other non-listed symptoms.

### Blood collection and sample processing

Blood was collected from all patients who were all negative for COVID-19 symptoms at the time of their visits. Blood specimens were collected in SST tubes, allowed to clot and centrifuged at 1100–1300 g for 20 min at room temperature. The specimens were aliquoted into sterile cryovials and stored immediately at – 80 °C until testing.

### SARS-CoV-2 antibody testing

Seropositivity was defined by a positive result using either the Emergency Use Authorization (EUA) Siemens Healthineers SARS-CoV-2 (COV2T) chemiluminescence-based assay, which measures total antibodies to RBD followed by a semi-quantitative assay for anti-RBD immunoglobulin (Ig)G.

### Analysis of cytokine data

Sera were tested by the Eve Technologies 71-Plex Discovery Assay (HD71) for human cytokines and chemokines. The procedure of variable cytokine selection, dimensionality reduction and clustering were performed using the R package Seurat. Cytokine production values were normalized using the function “ScaleData” in Seurat, underwent dimensionality reduction using the function “RunPCA”, and 5 principal components were identified for downstream analysis. The number of significant principal components to include in downstream analysis was determined based on the elbow point on the plot of standard deviations of principal components. Clusters were identified using the using the “FindNeighbors” function followed by “FindClusters” function with optimal resolution. UMAP was used for visualization purposes. Heatmap and violin plots were generated using the “DoHeatmap” and “VlnPlot” functions in Seurat. Distance-based redundancy analysis was used to determine the contribution of different factors to the variance observed within the patient cytokine profiles using the R package “vegan”. Bar graphs and dot plots were generated using the R package ggplot2. Statistical tests were selected based on appropriate assumptions with respect to data distribution and variance characteristics. Statistical differences in Fig. 2 and Supplemental Fig. 1 were calculated by Mann–Whitney U test by using the R package stats. To control for multiple testing, a false discovery rate (FDR) was calculated according to the Benjamini–Hochberg procedure using the R package "stats".

### Supplementary Information

Supplementary Figure 1.

## Data Availability

The data that support the findings of this study are available on request from the corresponding author (SYW). The data are not publicly available due to ethical considerations, including their containing information that could compromise the privacy of research participants.
