# Supplementary figures and images for: Cytokine signature in convalescent SARS-CoV-2 patients with inflammatory bowel disease receiving vedolizumab
Source: Sci Rep. 2024 Jan 2;14:186. doi: 10.1038/s41598-023-50035-1 (PMC10761911; doi:10.1038/s41598-023-50035-1)

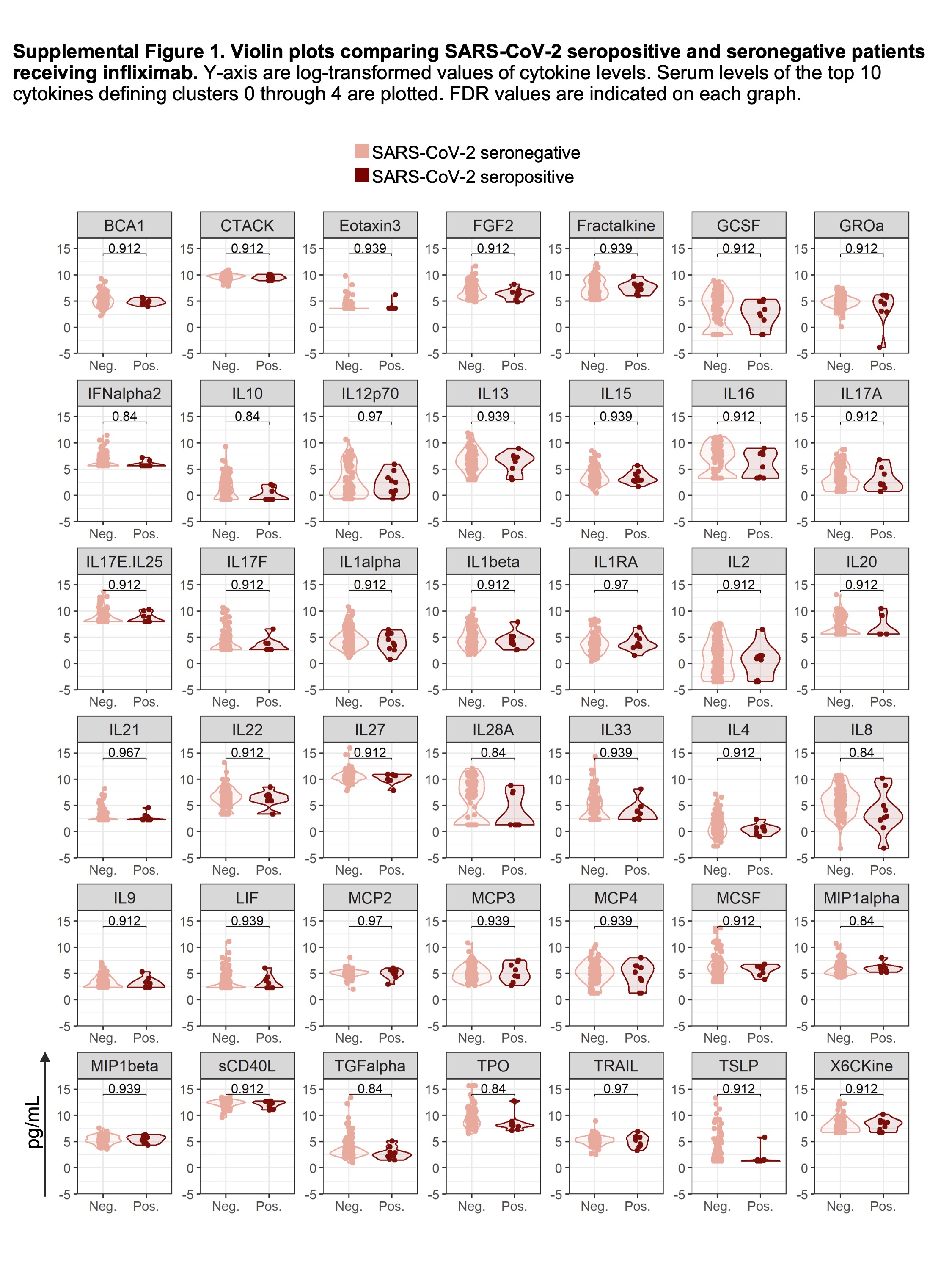

Supplement: Supplementary file 1 — Supplementary Figure 1. [file 41598_2023_50035_MOESM1_ESM.jpg]
